# Supplementary material for: Modeling of the chest wall response to prolonged bracing in pectus carinatum
Source: PLoS One. 2023 Aug 14;18(8):e0288941. doi: 10.1371/journal.pone.0288941 (PMC10424870; doi:10.1371/journal.pone.0288941)
Supplement: Attachment — Submitted filename: Response to Reviewers.docx [file pone.0288941.s001.docx]

# Response to Reviewers

**Journal Requirements:**

When submitting your revision, we need you to address these additional requirements.

1. Please ensure that your manuscript meets PLOS ONE's style requirements, including those for file naming. The PLOS ONE style templates can be found at 
<https://journals.plos.org/plosone/s/file?id=wjVg/PLOSOne_formatting_sample_main_body.pdf> and 
<https://journals.plos.org/plosone/s/file?id=ba62/PLOSOne_formatting_sample_title_authors_affiliations.pdf>

- Response: This has been reviewed and author contributions (previously missing) has been added.

2. We note that you have stated that you will provide repository information for your data at acceptance. Should your manuscript be accepted for publication, we will hold it until you provide the relevant accession numbers or DOIs necessary to access your data. If you wish to make changes to your Data Availability statement, please describe these changes in your cover letter and we will update your Data Availability statement to reflect the information you provide.

- Response: This is still the case as we are in the process of putting the data in an open-access institutional repository. The access information for the data will be provided when available.

3. Please include your full ethics statement in the ‘Methods’ section of your manuscript file. In your statement, please include the full name of the IRB or ethics committee who approved or waived your study, as well as whether or not you obtained informed written or verbal consent. If consent was waived for your study, please include this information in your statement as well.

- Response: This has been added to the first paragraph of the materials and methods section.

4. Please include a copy of Table 5 which you refer to in your text on page 5.

- Response: This was a typographical error and table 5 should be table 2. This change has been made.

5. We note you have included a table to which you do not refer in the text of your manuscript. Please ensure that you refer to Table 2 in your text; if accepted, production will need this reference to link the reader to the Table.

- Response: See response to comment 4 above.

6. Please review your reference list to ensure that it is complete and correct. If you have cited papers that have been retracted, please include the rationale for doing so in the manuscript text, or remove these references and replace them with relevant current references. Any changes to the reference list should be mentioned in the rebuttal letter that accompanies your revised manuscript. If you need to cite a retracted article, indicate the article’s retracted status in the References list and also include a citation and full reference for the retraction notice.

- Response: The reference list was reviewed and no changes were made.

**Reviewer Comments to the Author**

Reviewer #1: The manuscript is interesting and good designed and written
Introduction was good written
Materials and methods were good written
Results were good described
Discussion was good written and including the conclusion of the study

- Response: Thank you for you support of this work!

Reviewer #2: The authors presented a beautifully written manuscript on creating a model of chest wall response to prolonged bracing in pectus carinatum.

As the outcomes of bracing for pectus carinatum today is normally determined via clinical outcomes by the patient and provider, have the authors seen that their modeling method leads to quicker improvement of clinical outcomes secondary to patient compliance with bracing? What have the authors seen as the fail rate of their method that requires surgical intervention and at what time point did this occur? How could providers insure adequate patient compliance and logging of bracing hours?

This method helps predict bracing time but it could also be used to help determine operative vs non-operative treatment of pectus carinatum aka if it takes x number of bracing months to achieve good clinical response is that worthwhile from the patients' standpoint vs. proceeding immediately to surgical intervention. Congratulations to the authors on their hard work.

- Response: Thank you for your comments and suggestions. The following discussion on operative and non-operative approaches discussion for patients and providers has been added as well as discussion on future work in comparing the models to improved clinical outputs, fail rates, and patient compliance. These discussions were added to the “Discussion” section.
